# Pay Hospitals: Fiat Experimentum

**Published:** 1895-08-03

**Authors:** 


### Pay Hospitals: Fiat Experimentum.

Human nature is wondrously contradictory. In physiology, in therapeutics, in all matters which concern the patient more intimately than the physician, the doctor is all for experiment; and not for one experiment only, or for two or three, but for experiment piled upon experiment, planned and carried out by all manner of persons, under every conceivable set of conditions, if possible in every town and country in the world, until doubt is so completely killed and annihilated that it can never by any possibility hold up its head any more. Such is the latter-day medical man's high scientific standpoint. Theorising, *a priori* reasoning, the construction of temporary working hypotheses, all the methods of the pre-scientific ages of the world, he cannot tolerate for a moment; they are anathema to him.

By what perversity of elementary human nature does the medical practitioner swear allegiance to a totally different set of principles the moment his pocket begins to be concerned? In his resistance to the abuse of hospitals, both in their in-patient and their out-patient departments, we have over and over again proved ourselves to be entirely at one with him. In his opposition to pay wards as ordinarily conducted in this country we are also entirely on his side. But when we find that the mere mention of payment at hospitals is sufficient to put him into an unreasoning fury which is as foreign to science as snow is to summer, we confess to a feeling which is nothing short of amazement.

Is it possible that the pay ward question can ever be settled by mere see-saw, up and down, backward and forward argumentation? It is not possible. Moreover, the attempt to settle it in this unbusinesslike fashion reflects grave discredit upon the mental capacity alike of medical men and hospital managers. There is only one effectual method of solving the problem. *Fiat experimentum!* Once for all let an experiment be made. The very place for such an experiment is London. In our splendid metropolis we have every ideal facility for the making of such an experiment on the largest and most convincing scale. Patients of the right class are here in unlimited numbers. Scientific and rational physicians and surgeons are here. Hospital managers of the most competent business experience abound on every hand. A philanthropic public, eager for the best solution of this and many other problems, awaits such an experiment with sympathetic expectation. The general mind of all classes is open and prepared as it never was before for a rational attempt to deal adequately and permanently with one of the most important of the practical questions of the day. What hinders that an experiment should be made?

This is one of the proposals which the suggested conference should discuss. If the medical profession as a body would but be true to its own leading principle of competent experimentation, the thing would be done. Let us pull our mental faculties together; let us get rid of our unreasoning impatience; let us recognise that we are in the midst of an old civilisation, of abuses made venerable by age, of difficult and complex problems, of questions which not passion and impatience, but reason and philosophy must answer. We repeat, in this as in other matters, let us, as medical men, be true to the scientific principles in which we have been trained, and which, in any other department of our life, we have never known to fail. Let us bring the burning but never-ending question of pay hospitals and pay wards to the one proved and unfailing touchstone of "*Fiat experimentum.*"

